# Efficacy and tolerability of levocabastine and azelastine nasal sprays for the treatment of allergic rhinitis

**DOI:** 10.1155/S0962935195000780

**Published:** 1995-05

**Authors:** Ralph Mösges, Joachim Spaeth, Ludger Klimek

**Affiliations:** 1 Department of Otorhinolaryngology University of Aachen Aachen Germany; 2 Department of Otorhinolaryngology University of Mayence Mayence Germany

## Abstract

Levocabastine and azelastine are currently the only antihistamines available as nasal sprays for the topical therapy of seasonal allergic rhinitis. The present study was undertaken to compare the onset of action, efficacy and tolerability of these two agents in a total of 242 patients with this condition. This was an international, multicentre, open-label, randomized, parallel-group trial with 123 patients treated with levocabastine (0.5 mg/ml, two puffs per nostril twice daily) and 119 with azelastine (1 mg/ml, one puff per nostril twice daily). Onset of action was comparable for the two drugs with over 50% of patients in each group reporting significant symptomatic relief within 30 min of administration of the first dose of study medication. Therapeutic efficacy was also found to be comparable in the two groups with no statistically significant intergroup differences reported for any of the parameters evaluated, although assessments of global therapeutic efficacy revealed a trend favouring levocabastine. Levocabastine appeared to be better tolerated than azelastine (p = 0.06), with the incidence of the most common adverse experiences, application site reactions and taste disturbances, significantly higher on azelastine than with levocabastine (5% versus 1%; p = 0.05 and 5% versus 0%; p = 0.01, respectively). In conclusion, levocabastine nasal spray appears to be at least as effective as, but better tolerated than, azelastine nasal spray for the treatment of seasonal allergic rhinitis.


Mediators of Inflammation 4, S11–S15 (1995)

### Efficacy and tolerability of levocabastine and azelastine nasal sprays for the treatment of allergic rhinitis

Ralph Mösges,[1,CA] Joachim Spaeth[1] and Ludger Klimek[2]

[1]Department of Otorhinolaryngology, University of Aachen, Aachen, Germany;
[2]Department of Otorhinolaryngology, University of Mayence, Mayence, Germany

[CA]Corresponding Author

**LEVOCABASTINE and azelastine are currently the only antihistamines available as nasal sprays for the topical therapy of seasonal allergic rhinitis. The present study was undertaken to compare the onset of action, efficacy and tolerability of these two agents in a total of 242 patients with this condition. This was an international, multicentre, open-label, randomized, parallel-group trial with 123 patients treated with levocabastine (0.5 mg/ml, two puffs per nostril twice daily) and 119 with azelastine (1 mg/ml, one puff per nostril twice daily). Onset of action was comparable for the two drugs with over 50% of patients in each group reporting significant symptomatic relief within 30 min of administration of the first dose of study medication. Therapeutic efficacy was also found to be comparable in the two groups with no statistically significant intergroup differences reported for any of the parameters evaluated, although assessments of global therapeutic efficacy revealed a trend favouring levocabastine. Levocabastine appeared to be better tolerated than azelastine ($p = 0.06$), with the incidence of the most common adverse experiences, application site reactions and taste disturbances, significantly higher on azelastine than with levocabastine (5% versus 1%; $p = 0.05$ and 5% versus 0%; $p = 0.01$, respectively). In conclusion, levocabastine nasal spray appears to be at least as effective as, but better tolerated than, azelastine nasal spray for the treatment of seasonal allergic rhinitis.**

**Key words:** Allergic rhinitis, Azelastine, Levocabastine, Topical antihistamines

### Introduction

In recent years there has been renewed interest in the topical treatment of allergic rhinoconjunctivitis. A topical agent may be expected to have a number of advantages over an orally administered drug including a more rapid onset of action, since the active agent is administered directly to the affected site, and a reduced potential for unwanted systemic reactions. To date, only two antihistamines are available for the topical treatment of seasonal allergic rhinitis, levocabastine and azelastine.

Levocabastine has been specifically developed for topical use,[1] and is available as both eye drops and nasal spray. *In vitro* studies have demonstrated that it is the most potent antihistamine available to date,[2] being 1000 times more potent than azelastine in the compound 48/80 lethality test in rats.[3] Comparative clinical trials have shown topically applied levocabastine to be well tolerated and at least as effective as the oral antihistamines terfenadine and loratadine for the treatment of seasonal allergic rhinoconjunctivitis,[4–7] with statistically significant differences in favour of the topical drug observed in some studies.[4,5] In contrast, azelastine has primarily been developed as an anti-asthma agent,[8] and is only available as a nasal spray. In addition to its antihistaminic activity, this agent appears to have other anti-allergic properties which may be of clinical benefit to patients with allergic rhinitis. In clinical trials, intranasal azelastine has been shown to be as effective as oral terfenadine, astemizole and loratadine for the treatment of patients with this allergic disorder.[9,10]

As no direct comparison of these two topical antihistamines has been undertaken to date, the present study was initiated to compare the efficacy, onset of action and tolerability of levocabastine and azelastine nasal sprays in patients with seasonal allergic rhinitis. The study was open-label, rather than blinded, as with a nasal spray the design of the delivery device may influence therapeutic efficacy. Furthermore, the levocabastine and azelastine nasal sprays

© 1995 Rapid Communications of Oxford Ltd commercially available deliver different volumes necessitating different dosage regimens. Consequently, topical placebos would be required to fully blind treatment. Response rates as high as 35% have been reported with placebo nasal sprays which may distort any true differences in therapeutic efficacy between the two treatment groups.[11]

### Materials and Methods

*Patients:* Patients aged between 12 and 70 years with at least a 1-year history of seasonal allergic rhinitis severe enough to require anti-allergic therapy, together with a positive skin prick and/or (radioallergosorbent) RAST test for grass pollen were eligible for entry into this trial. In addition, patients were required to exhibit at least two typical nasal symptoms of moderate severity at the time of entry into the trial. The trial was performed between 1 May 1993 and 15 August 1993 at the height of the grass pollen season.

Exclusion criteria included: concurrent diseases which could interfere with assessment of the study drugs such as vasomotor rhinitis, rhinitis medicamentosa, active infective sinusitis, upper respiratory tract infections and large obstructive nasal polyps; concomitant therapy with any medication that might interfere with the assessment of the study drugs with a washout period of 1 month for systemic corticosteroids, 2 weeks for topical corticosteroids and sodium cromoglycate, 1 week for all antihistamines with the exception of astemizole for which a washout period of 6 weeks was required, and 3 days for decongestants and all other ocular and nasal medication; concurrent hyposensitization therapy; and use of an investigational drug within 30 days prior to entry into the trial. Patients with evidence of major organ disease, and pregnant or nursing women were also excluded from participation in the study.

*Study design:* This was an international (Belgium, Germany), multicentre, open-label, parallel-group trial. Although the study was open, treatment was randomized and the two antihistamine sprays were supplied in identical packaging. Patients received either levocabastine nasal spray (0.5 mg/ml) two puffs per nostril twice daily or azelastine nasal spray (1 mg/ml) one puff per nostril twice daily. Treatment duration was 1 week. The study was conducted in accordance with the Declaration of Helsinki and subsequent revisions. The study protocol was approved by the local ethics committee and all patients gave informed consent, with informed parental consent for patients under 18 years of age recruited for the Belgian part of the study

*Assessments and evaluations:* The severity of sneezing, rhinorrhoea, nasal itching, nasal congestion and concurrent ocular symptoms was assessed by the patients on a daily basis and by the investigator at the start of the trial, after 1 week of treatment and at the study end-point, using a 4-point scale (0 = absent, 1 = mild [noticeable on occasion but not bothersome], 2 = moderate [noticeable from time to time and tended to be bothersome], 3 = severe [frequent and bothersome]). Patients also assessed the overall severity of their rhinitis on a daily basis using a 100 mm visual analogue scale (VAS; extremes; 0 = absent, 100 = very severe). In addition, both the patients and investigator performed global evaluations of treatment efficacy at the end of the trial rating the effect of therapy as excellent, good, moderate or poor.

To permit evaluation of onset of action, patients were asked to record the overall severity of rhinitis symptoms on a diary card using a 100 mm visual analogue scale (VAS; extremes: 0 = absent, 100 = very severe) immediately before taking the first dose of study medication and then at 5, 10, 20, 30 and 40 min, and 1, 2, 3, 4, 6 and 8 h after administration. In addition, patients were requested to indicate how soon they felt significant improvement in rhinitis symptom severity following administration of the first dose of study medication.

Any adverse events experienced during the trial were recorded by the patients in their diaries. Pollen counts were performed daily at each centre. Pollen counts greater than 50 pollen grains/$mm^3$ were classed as high.

*Statistical analysis:* An intention-to-treat analysis was performed. Onset of action was derived from the patients' diary data generated following administration of the first dose of study medication. In addition to the individual symptoms listed above, the sum of all nasal symptoms and the sum of all symptoms were calculated and analysed at the start of the trial and at the end of the 1-week treatment period. Daily VAS ratings of symptom severity, the area under the curve (AUC) for the different VAS scores (expressed as a percentage of the maximal AUC and calculated using the trapezoidal rule), the percentage of days with severe symptoms (VAS $\geqslant$ 50%) and the percentage of symptom-free days (VAS $\leqslant$ 10%) were also determined for all parameters recorded in the patients' diaries. The influence of the pollen count on the daily symptom scores was also evaluated. All intergroup differences

**Table 1.** Patient demographics

| | Levocabastine | Azelastine |
|---|---|---|
| Number of patients | 123 | 119 |
| (male/female) | (55/68) | (66/53) |
| Median age in years | 30.0 | 30.0 |
| (range) | (12–65) | (12–69) |
| Median weight in kg | 67.0 | 68.0 |
| (range) | (33–105) | (36–133) |

were subjected to analysis of variance testing (ANOVA).

### Results

A total of 242 patients were entered into this trial, 123 of whom received levocabastine and 119 azelastine. Patient demographics are shown in Table 1. In all, 17 patients withdrew from the trial (six levocabastine-treated patients and eleven of those on azelastine). Reasons for withdrawal are summarized in Table 2. Adverse experiences resulting in withdrawal included dry throat (levocabastine), cough (azelastine) and dyspnoea with wheezing (azelastine). As shown in Table 3, baseline symptom severity was generally comparable in the two treatment groups, although total symptom severity was found to be 7% higher in the azelastine treatment group than in the group which received levocabastine at the start of the trial ($0.05 < p < 0.1$).

**Table 2.** Reasons for withdrawal in the two treatment groups

| | Levocabastine ($n$ = 123) | Azelastine ($n$ = 119) |
|---|---|---|
| Number of patients | 6 | 11 |
| Reason* | | |
| Adverse experience | 1 | 3 |
| Insufficient response | 2 | 4 |
| Lost to follow-up | 3 | 1 |
| Lack of symptoms | | 1 |
| Uncooperative | | 2 |
| Other | | 2 |

*Some patients gave more than one reason for withdrawal.

**Table 3.** Investigator assessments of symptom severity[a] in the two treatment groups at baseline and at the end of the trial

| | Levocabastine | | Azelastine | |
|---|---|---|---|---|
| | Baseline | Endpoint | Baseline | Endpoint |
| Sneezing | 2.2 | 0.7 | 2.3 | 0.7 |
| Rhinorrhoea | 2.2 | 0.8 | 2.4 | 0.8 |
| Nasal itching | 1.8 | 0.6 | 1.8 | 0.6 |
| Nasal congestion | 1.9 | 1.0 | 2.1 | 1.1 |
| Ocular symptoms | 1.7 | 0.6 | 1.8 | 0.6 |
| Total nasal symptoms | 8.1 | 3.1 | 8.6 | 3.2 |
| Total all symptoms | 9.8 | 3.6 | 10.5* | 3.8 |

[a]Each symptom was assessed using a 4-point scale (0 = absent, 1 = mild, 2 = moderate, 3 = severe).
*$0.05 < p < 0.1$.

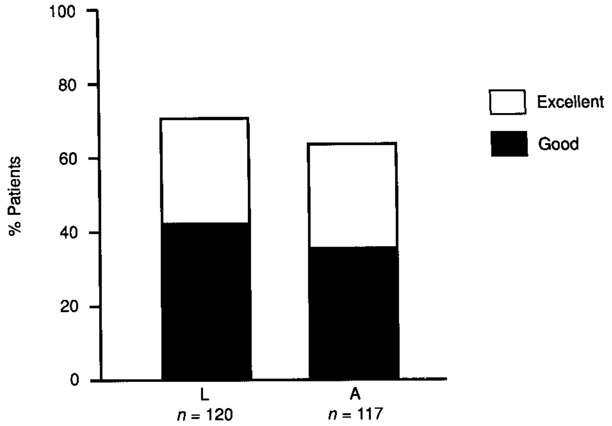

FIG. 1. Investigator evaluation of global therapeutic efficacy at the end of the 1-week treatment period. L = levocabastine, A = azelastine.

Pollen concentrations were considered sufficient to elicit symptoms of seasonal allergic rhinitis in both countries throughout the trial period with high counts recorded on 79% of study days.

Assessments of global therapeutic efficacy were found to favour levocabastine, however intergroup differences did not attain statistical significance. At the end of the trial, the investigator rated therapeutic efficacy as excellent or good in 70% of levocabastine-treated patients compared with 63% of those who received azelastine (Fig. 1). The corresponding values for the patients' assessments were 68% and 61% in the two groups, respectively.

Both the investigators' and patients' assessments revealed that levocabastine and azelastine provided comparable reduction of symptom scores for sneezing, rhinorrhoea, nasal itching, nasal congestion and concurrent ocular symptoms, as well as total symptom severity (Table 3, Figs 2 and 3), with a similar percentage of

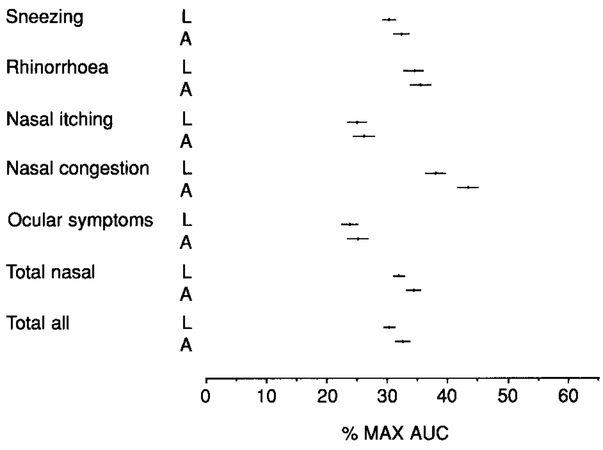

FIG. 2. Patient evaluations of symptom severity during the 1-week trial expressed as a percentage of the maximum AUC for each variable (± SEM).

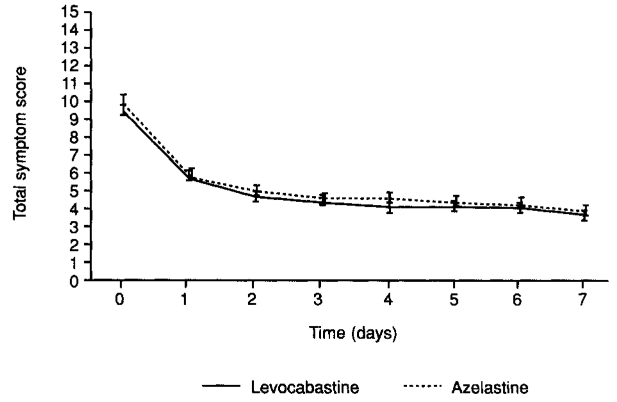

FIG. 3. Patient evaluations of symptom severity during the 1-week trial. Day-by-day score for all symptoms combined (mean ± SEM).

Table 4. Adverse experiences occurring in at least two patients per treatment group

| | Levocabastine ($n$ = 120) | | Azelastine ($n$ = 118) | |
|---|---|---|---|---|
| | $n$ | % | $n$ | % |
| Number of patients reporting an adverse experience | 13 | 10.8 | 23 | 19.5 |
| Adverse experience | | | | |
| Application site reaction | 1 | 0.8 | 6 | 5.1 |
| Taste disturbance | – | – | 6 | 5.1 |
| Rhinitis | 2 | 1.7 | 3 | 2.5 |
| Headache | 3 | 2.5 | 1 | 0.8 |
| Fatigue | 2 | 1.7 | 2 | 1.7 |
| Injury | – | – | 3 | 2.5 |
| Pharyngitis | 2 | 1.7 | 1 | 0.8 |
| Bronchospasm | – | – | 2 | 1.7 |
| Abdominal pain | 2 | 1.7 | – | – |

symptom-free days in the two treatment groups (7% on levocabastine compared with 9% on azelastine). Analysis as a function of pollen count also failed to reveal any significant intergroup differences in therapeutic efficacy on days with high pollen counts.

Onset of action was found to be comparable for the two drugs as assessed by patients' VAS ratings following administration of the first dose of study medication. More than half the patients in each treatment group reported relief of symptoms within 30 min of drug application (53% on levocabastine and 54% on azelastine). Relief from symptoms was found to be maintained at 8 h after administration of the first dose of study medication in both treatment groups.

The incidence of adverse experiences was found to be higher on azelastine than levocabastine, with adverse reactions reported by 19% and 11% of patients in the two groups respectively

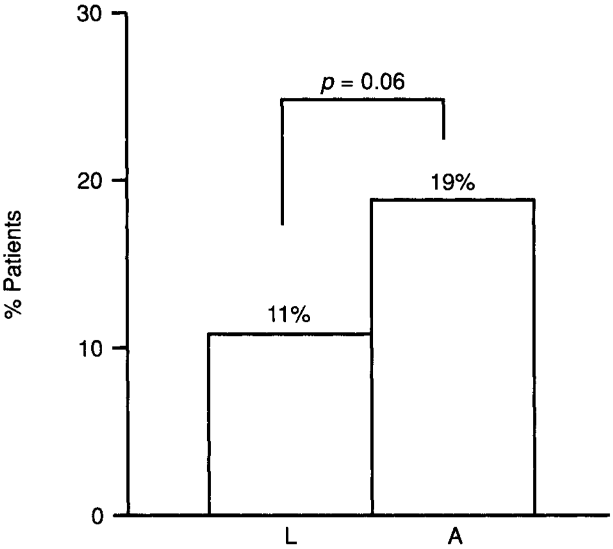

FIG. 4. Percentage of patients reporting adverse experiences in the two treatment groups.

($p$ = 0.06) (Fig. 4). As shown in Table 4, the most commonly reported adverse events were application site reactions and taste disturbances. These were significantly more frequent in the azelastine group than in the group treated with levocabastine, with application site reactions reported by 5% of patients on azelastine compared with 1% of those who received levocabastine ($p$ = 0.05) and taste disturbances by 5% and 0% of patients in the two groups, respectively ($p$ = 0.01). No significant intergroup differences in frequency were apparent for any of the other common adverse reactions reported.

### Discussion

The results of this study suggest that levocabastine and azelastine nasal sprays have comparable efficacy in patients with seasonal allergic rhinitis with both agents providing rapid, effective and sustained symptomatic relief. Although both the investigators' and the patients' assessments of global therapeutic efficacy revealed a trend in favour of levocabastine, no statistically significant intergroup differences were seen for any of the efficacy parameters evaluated. This is in keeping with the results of previous studies which have shown that these two topical antihistamines are at least as effective as oral $H_1$-receptor antagonists for the treatment of this common condition.[4–7,9]

The effects of levocabastine and azelastine were comparable on all symptoms of allergic rhinitis, including nasal congestion. Nasal congestion is generally less responsive to treatment with antihistamines, although it has been claimed that azelastine is particularly effective for the treatment of this symptom due to its anti-inflammatory properties.[12] However, the results of the present study suggest the effect of levocabastine on nasal congestion is at least as good as that seen with azelastine.

In this trial, levocabastine nasal spray was clearly better tolerated with the overall incidence of adverse experiences markedly higher in azelastine-treated patients than in those who received levocabastine (19% versus 11%; $p$ = 0.06). As might be expected from the route of drug administration, application site reactions and taste disturbances were the most frequent adverse events occurring during the course of the trial. However, the incidence of these reactions was found to be significantly greater in patients on azelastine than with levocabastine (5% versus 1% for application site reactions; $p$ = 0.05 and 5% versus 0% for taste disturbances; $p$ = 0.01, respectively).

Topical antihistamines such as levocabastine and azelastine represent a significant advance in antihistamine research and provide a useful alternative to oral antihistamines as a therapeutic option for the treatment of patients with allergic rhinitis. Results of this initial comparative study suggest that the two agents have similar therapeutic efficacy, but that levocabastine nasal spray is better tolerated. Coupled with the fact that this agent is also available as eye drops for the relief of concurrent ocular symptoms, these findings suggest that levocabastine may be the preferred topical antihistamine for the treatment of allergic rhinoconjunctivitis.

### References

1. Awouters F, Niemegers C, Janssen T, *et al.* Levocabastine: pharmacological profile of a highly effective inhibitor of allergic reactions. *Agents Actions* 1992; **35:** 12.
2. Van Wauwe JP. Animal pharmacology of levocabastine: a new type of $H_1$-antihistamine well-suited for topical application. In: Mygind N, Naclerio RM, eds. *Rhinoconjunctivitis: New Perspectives in Topical Treatment of Seasonal Allergic Rhinitis.* Proceedings of the XIIIth International Congress of Allergology and Clinical Immunology. Gottingen: Hogrefe and Huber, 1989; 27–34.
3. Megens AAHP, Vermeire J, Awouters FHL. Protection from compound 48/80-induced shock in rats: comparison of nine recent antihistamines with levocabastine. Life Science Advances. *Pharmacology* 1995; in press.
4. Søhoel P, Freng BA, Kramer J, *et al.* Topical levocabastine compared with oral terfenadine for the treatment of seasonal allergic rhinoconjunctivitis. *J Allergy Clin Immunol* 1993; **92:** 73–81.
5. Bahmer FA, Ruprecht KW. Safety and efficacy of topical levocabastine compared with oral terfenadine. *Ann Allergy* 1994; **72:** 429–434.
6. The Livostin Study Group. A comparison of topical levocabastine and oral terfenadine in the treatment of allergic rhinoconjunctivitis. *Allergy* 1993; **48:** 519–524.
7. The Swedish GP Allergy Team. Topical levocabastine compared with oral loratadine for the treatment of seasonal allergic rhinoconjunctivitis. *Allergy* 1994; **49:** 611–615.
8. McTavish D, Sorkin EM. Azelastine. A review of its pharmacodynamic and pharmacokinetic properties and therapeutic potential. *Drugs* 1989; **38:** 778–800.
9. Janssens MM-L, Howarth PH. The antihistamines of the nineties. *Clin Rev Allergy* 1993; **11:** 111–153.
10. Mösges R, Dostal M, *et al.* Ist bei der allergischen Rhinitis die Therapie mit Azelastin Nasenspray wirkungsäquivalent der Systemischen Therapie mit Loratadin-Tabletten? In: Mösges R, Schlöndorff G (eds). *Topische Therapie der allergischen Rhinitis.* Zülpich, Germany: Biermann Verlag, 1993.
11. Dechant KL, Goa KL. Levocabastine: a review of its pharmacological properties and therapeutic potential as a topical antihistamine in allergic rhinitis and conjunctivitis. *Drugs* 1991; **41:** 202–224.
12. Thomas KE, Ollier S, Ferguson H, Davies RJ. The effect of intranasal azelastine, Rhinolast®, on nasal airways obstruction and sneezing following provocation testing with histamine and allergen. *Clin Exp Allergy* 1992; **22:** 642–647.

ACKNOWLEDGEMENTS. The authors would like to acknowledge the assistance of members of the Janssen Research Group of General Practitioners in Belgium: B. Bauvignet, L. Berghmans, P. Berwouts, J. Boxus, N. Buyse, J. Croo, P. Cybulski, W. De Graeve, R. De Jongh, H. de Meester, L. De Waele, A. Dernovoi, J. Detiège, J. Dewachter, P. Dewaele, P. Duerinckx, H. Floré, M. Heylbroek, R. Jacquemyns, P. Jensen, P. Leemans, M. Lootens, J. Merlin, G. Metz, J. Meurant, J. Missant, J. Moerman, J. Mortelmans, C. Paelinck, G. Peeters, Y. Pil, M. Raes, N. Soumoy, W. Spaepen, F. Surmont, J. Van Baelen, E. Van Den Bossche, E. Van Der Meersche, C. Van Der Mullen, F. Van Dessel, J. Van Heirstraeten, M. Vanbellinghen, J. Vanleeuwe, D. Vantroyen, J. Verhaeghe, L. Vleugels; and of the following ENT experts and General Practitioners in Germany: R. Modi, F. Peters, M. Sondermann.
